# Diagnostic and prognostic utilities of humoral fibulin-3 in malignant pleural mesothelioma: Evidence from a meta-analysis

**DOI:** 10.18632/oncotarget.14712

**Published:** 2017-01-18

**Authors:** Dongxu Pei, Yongwei Li, Xinwei Liu, Sha Yan, Xiaolan Guo, Xiaona Xu, Xiaoxia Guo

**Affiliations:** ^1^ Department of Clinical Laboratory, Hennan Province Hospital of TCM, Zhengzhou, Henan, China; ^2^ Department of Clinical Laboratory, Anyang Hospital of Traditional Chinese Medicine

**Keywords:** fibulin-3, malignant pleural mesothelioma, diagnosis, prognosis, biomarker

## Abstract

Fibulin-3 has emerged as a promising novel biomarker in conforming or monitoring malignant pleural mesothelioma (MPM). This study sought to evaluate the diagnostic and prognostic efficacies of humoral fibulin-3 for MPM. Seven eligible publications comprising 468 MPM cases for diagnosis, and 138 for prognosis were identified. Results manifested that humoral fibulin-3 sustained a pooled sensitivity of 0.62 (95% CI: 0.45–0.77) and specificity of 0.82 (95% CI: 0.73–0.89) in discriminating MPM patients from cancer-free individuals, corresponding to an AUC (area under the curve) of 0.81. For the survival analysis, fibulin-3 expression was not markedly associated with overall survival (OS) time of the MPM patients [HR (hazard ratio): 1.84, 95% CI: 0.75–4.56, *P* = 0.185]. In the subgroup analyses stratified by test matrix and ethnicity, data revealed that serum-based fibulin-3 examination achieved superior accuracy than plasma-based analysis (sensitivity: 0.77 versus 0.54; specificity: 0.85 versus 0.77; AUC: 0.92 versus 0.69); additionally, testing of fibulin-3 in Europeans retained higher efficacy than those in Americans and Australians. Taken together, fibulin-3 confers a relatively high diagnostic efficacy and is acceptable to be an auxiliary biomarker to aid in MPM identification.

## INTRODUCTION

Malignant pleural mesothelioma (MPM) is a universally fatal neoplasm with high lethality features of easy metastasis and poor clinical outcomes [1–4]. The etiology of MPM is strongly linked to previous exposure to asbestos, although other aetiopathogenetic factors could not be eliminated [5, 6]. Despite the new advantages in therapeutics, the median survival time of patients with MPM remains dissatisfying, which only ranged from 6–18 months following diagnosis [3, 6]. Timely diagnosis of MPM is limited by the long latency stage in clinic, on this point, developing reliable diagnostic and/or prognostic markers for MPM will significantly benefit and enhance the clinical care.

Human fibulin-3, encoded by the epidermal growth factor-containing fibulin-like extracellular matrix protein-1 (EFEMP-1) gene, is identified as a secreted glycoprotein and plays an essential role in regulation of cell proliferation and migration [7, 8]. Recent findings have documented the significantly altered expression status of humoral fibulin-3 in mesothelioma, thereby highlighting its promising application as a novel biomarker for MPM diagnosis and prognosis [9–18]. Nevertheless, single study often presented with inaccurate and insufficient information due to restrictions from limited sample size and research programs. For instance, a study from Pass et al held that fibulin-3 was adequately sensitive and specific in confirming MPM, with a diagnostic sensitivity and specificity up to 90% [12]. However, some research reported that fibulin-3 testing only yielded a diagnostic sensitivity from 12.7% to 59%, and therefore questioned the application of fibulin-3 as a marker for MPM [10, 15]. Upon the above arguments, we conducted a comprehensive meta-analysis and aimed to evaluate the clinical utility, including the diagnostic and prognostic capabilities of humoral fibulin-3 for MPM.

## RESULTS

### Study features and article quality

**Figure 1:**
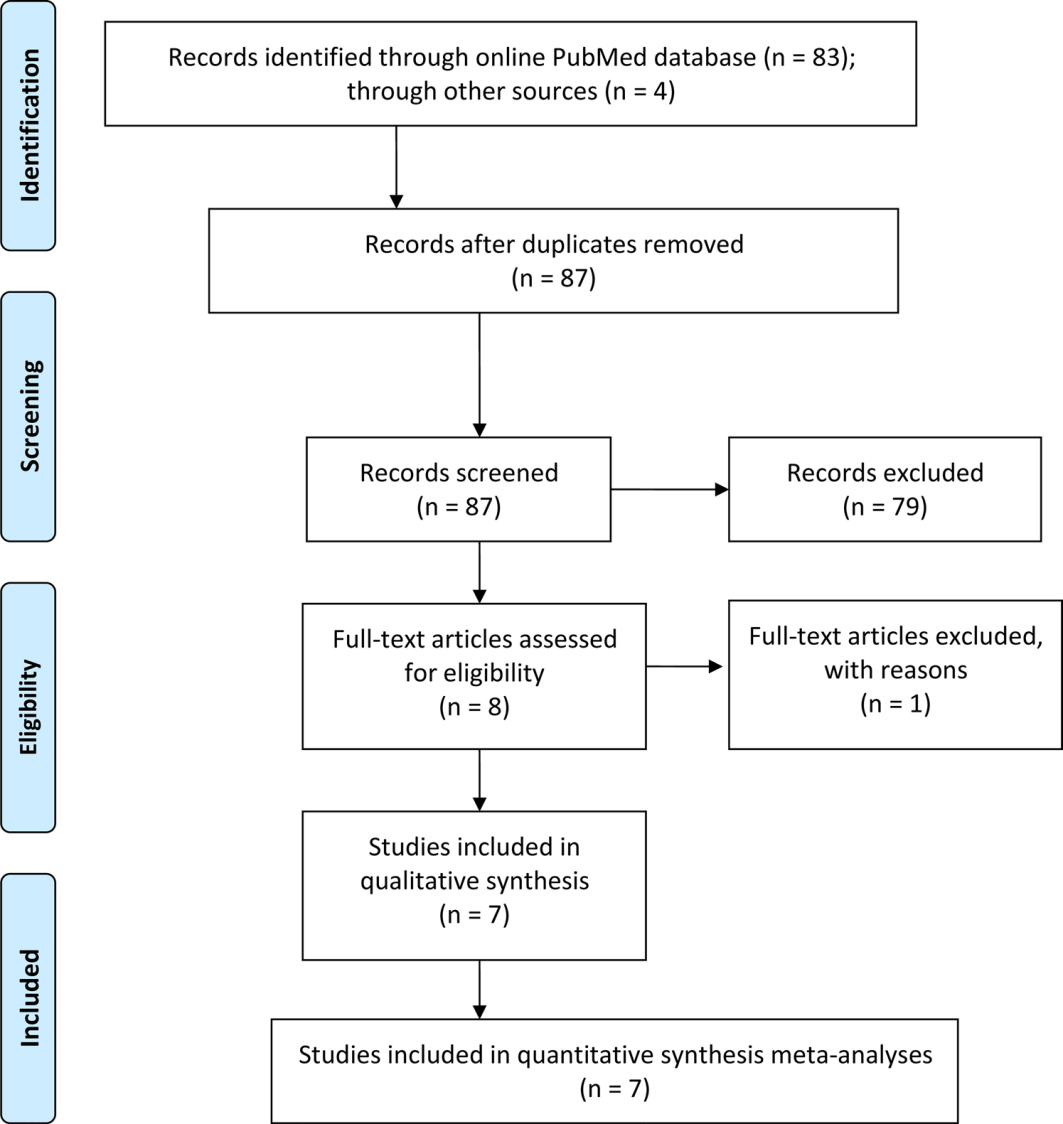
Flow diagram of the study enrollment procedure

According to the predefined criteria, 83 citations were obtained from the online PubMed database, yet 4 records were obtained through a search of article references. As shown in Figure 1, 79 records were excluded either due to the status of reviews, basic research articles or the contents were unrelated to fibulin-3 diagnosis or prognosis. Eight eligible studies then received full text evaluation, and in one of them, the ROC curve analysis compared the status of fibulin-3 in monitoring chemotherapy response of MPM, data from which were eventually eliminated [19]. Last, 6 studies for diagnosis [9, 10, 12, 14, 15, 17], and 3 for prognosis [10, 15, 16], were enrolled for the meta-analysis.

The main characteristics of each study are summarized in Table 1. A total of 468 MPM patients and 664 matched controls for the diagnostic analysis, and 138 MPM cases for prognostic category were obtained. All patients with MPM were prior to treatment and the final diagnosis all relied on the histopathological examinations. The control sources comprised healthy participants, asbestos-exposed individuals and non-MPM related effusions. The specimen type involved plasma, serum and effusion, and abundance of fibulin-3 was determined by enzyme linked immunosorbent assay (ELISA) in humoral. Study population consisted of Australians, Americans and Europeans. In the survival analysis, the primary endpoint of overall survival (OS) was extracted and the follow-up time was from 12 to 20 months.

**Table 1:** Main features of the included studies for humoral fibulin-3 in identification of MPM

Author	Year	Area	Case vs. Control type	Asbestos exposure (%) or years	Patient vs. Control size	Test method	Sample type	Cut-off value (ng/ml)
Kaya et al. [9]	2015	Turkey	MPM vs. Normal	83.7%	43 vs.40	ELISA	Serum	30.1
			MPM vs. Normal	83.7%	43 vs.40	ELISA	Serum	36.6
			MPM vs. Normal	83.7%	43 vs.40	ELISA	Serum	48.8
Creaney et al. [10]	2014	Australia	MPM vs. Non-MPM	Unclear	82 vs.120	ELISA	Plasma	29.0
			MPM vs. Non-MPM	Unclear	82 vs.121	ELISA	Plasma	52.0
			MPM vs. Non-MPM	Unclear	103 vs.71	ELISA	Pleural effusion	346.0
Pass et al. [12]	2012	America	MPM vs. Non-MPM	52.3 or 95.8%	92 vs.290	ELISA	Plasma	52.8
			MPM vs. Non-MPM	52.3 or 95.8%	92 vs.136	ELISA	Plasma	52.8
			MPM vs. Non-MPM	52.3 or 95.8%	92 vs.8	ELISA	Plasma	67.1
			MPM vs. Non-MPM	52.3 or 95.8%	92 vs.22	ELISA	Plasma	66.6
			MPM vs. Non-MPM	52.3 or 95.8%	92 vs.30	ELISA	Plasma	67.1
			MPM vs. Non-MPM	52.3 or 95.8%	92 vs.259	ELISA	Plasma	44.4
Demir et al. [17]	2015	Turkey	MPM vs. Normal	42.9 ± 19.1 years	42 vs.41	ELISA	Sera	51.4
Napolitano et al. [14]	2016	America	MPM vs. Normal	Unclear	22 vs.20	ELISA	Serum	Unclear
			MPM vs. Non-MPM	Unclear	22 vs.25	ELISA	Serum	Unclear
Kirschner et al. [15]	2015	Australia	MPM vs. Non-MPM	Unclear	37 vs.32	ELISA	Plasma	29.0
			MPM vs. Non-MPM	Unclear	47 vs.24	ELISA	Plasma	29.0

MPM: malignant pleural mesothelioma; ELISA: enzyme linked immunosorbent assay, vs: versus.

**Figure 2:**
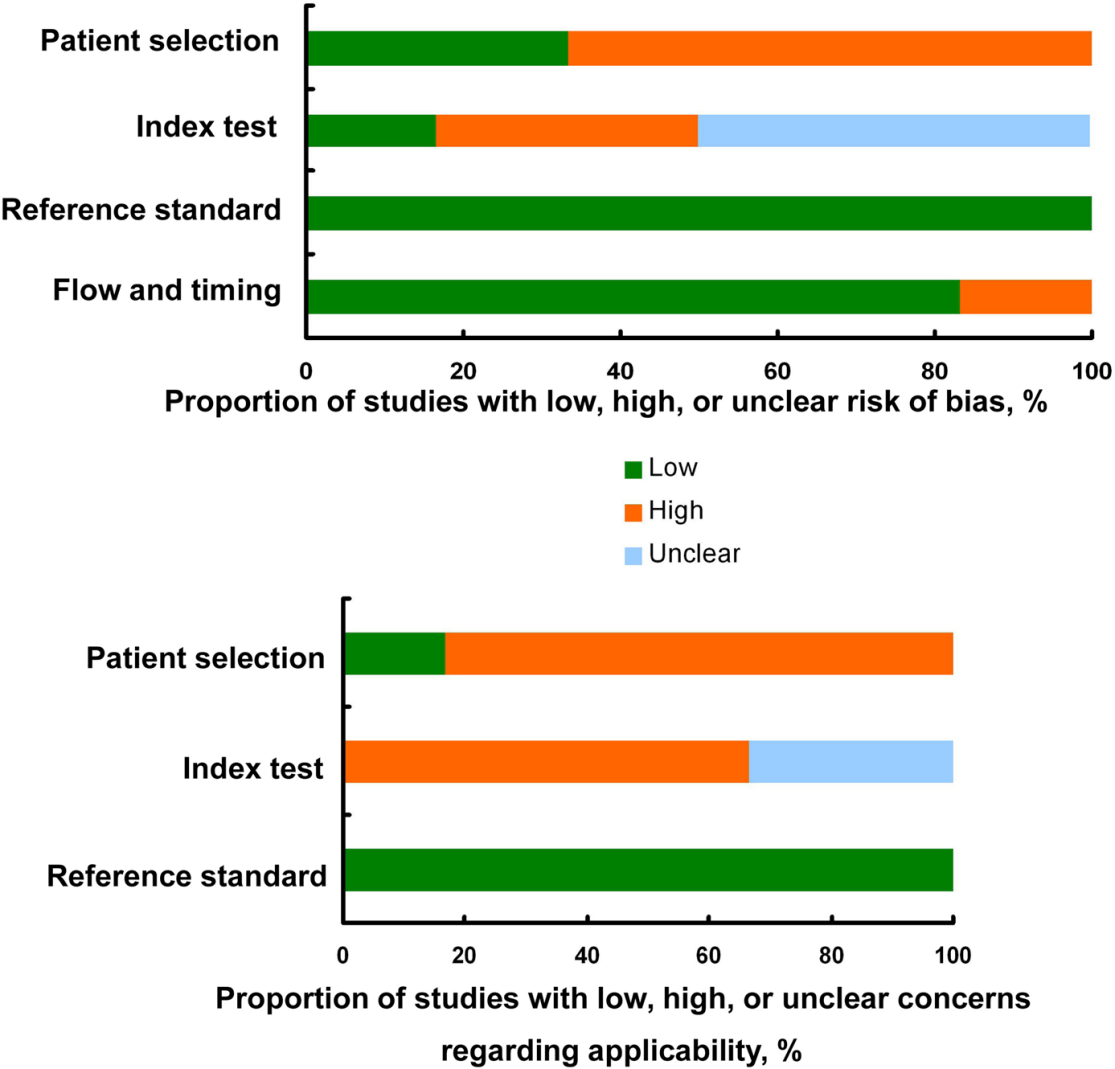
Study quality assessed by the QUADAS II checklist

The included cohorts were evaluated by the QUADAS II and Newcastle-Ottawa Scale (NOS) checklists [20, 21]. Figure 2 indicated the proportions of diagnostic studies with low, high, or unclear concerns regarding risk of bias and applicability by QUADAS II list. Scores of the prognostic studies judged by the NOS checklist were listed in Supplementary Table 1.

### Heterogeneity

Exploring of study heterogeneity was enabled by the testing of threshold and non-threshold effects among studies [22]. As exemplified in Table 2, Spearman's correlation coefficient of the overall pooled diagnostic analysis after adjustment was estimated to be 0.254, with a *P* value of 0.362, hinting that no significant heterogeneity was generated due to threshold effect. On the other hand, Cochran's-Q and I^2^ tests were performed to analyze heterogeneity from non-threshold effect, wherein, heterogeneity appeared in the overall pooled analysis after adjustment (Cochran's-Q = 80.06, *P* = 0.0000, I^2^ = 82.5%), as well as in the stratified analyses (Table 2). For the prognostic analysis, the I^2^ was estimated to be 87.3%, also revealing a significant heterogeneity in studies. As a result, a random effect model was finally employed for the combined analyses.

**Table 2:** Heterogeneity analysis of the diagnostic studies by Meta-disc 1.4 software

Analysis	Spearman correlation Coefficient	Cochran's-Q test	I^2^ test (%)	Heterogeneity	
	*P* value	*P* value		Threshold effect	Non-threshold effect
Overall ( adjusted )	0.362	0.0000	82.5	No	Yes
Overall (un-adjusted)	0.573	0.0000	88.2	No	Yes
Semen type					
Plasma	0.015	0.0003	74.1	Yes	Yes
Serum	0.674	0.0038	71.2	No	Yes
Ethnicity					
Australian	0.000	0.1866	37.6	Yes	No
American	0.354	0.0004	77.9	No	Yes
European	1.000	0.0012	81.1	No	Yes

### Influence analysis and diagnostic accuracy

**Figure 3:**
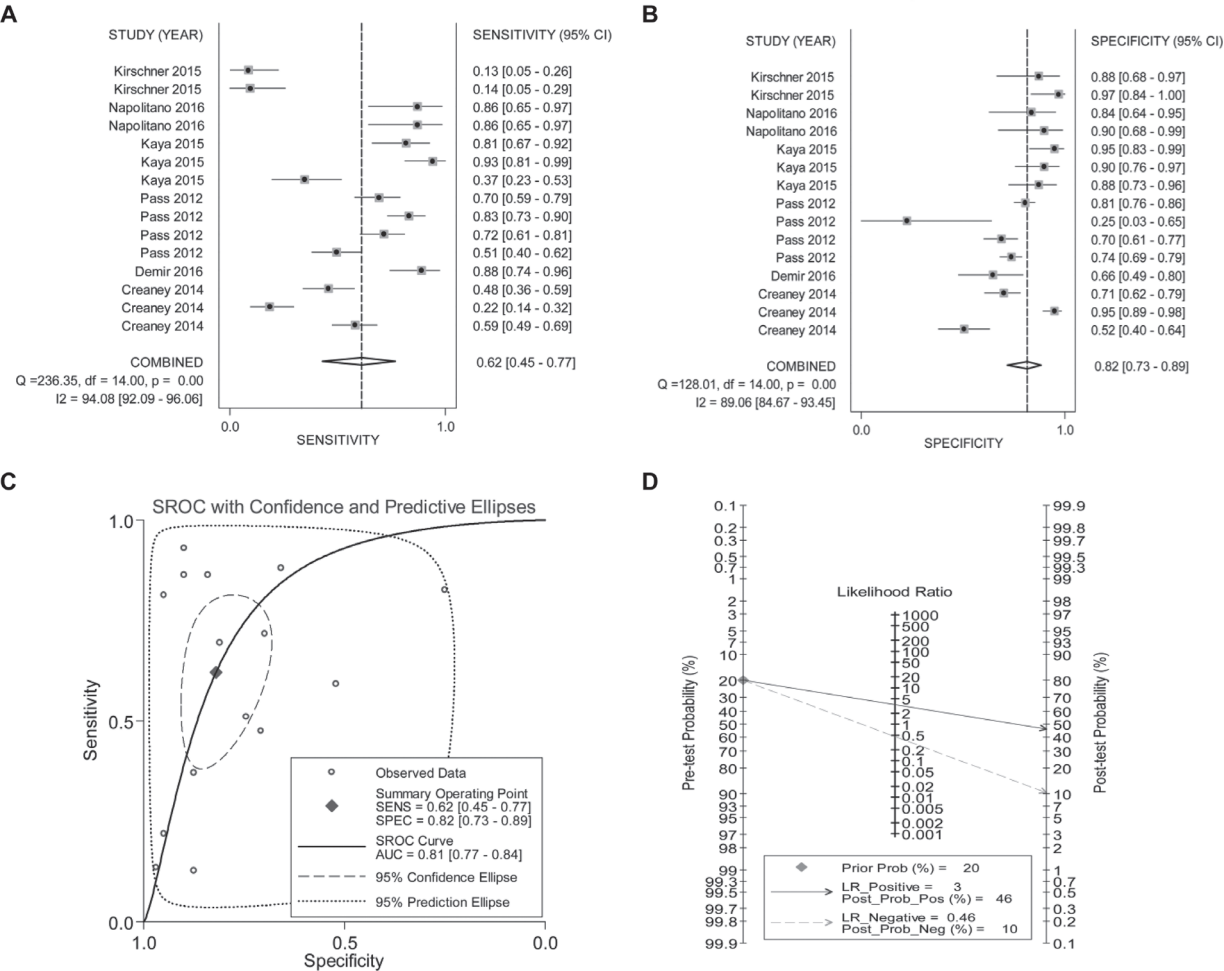
Forest plots of (**A**) pooled sensitivity, (**B**) specificity, (**C**) SROC curve and (**D**) post-test probability for humoral fibulin-3 in confirming MPM.

As the existence of significant heterogeneity among studies which may further compromise the overall accuracy of pooled analyses, we conducted influence analysis to deeply trace the outlier studies. Data showed that 2 individual studies were evaluated as outliers and were finally eliminated (Supplementary Figure 1). Accordingly, the adjusted meta-analyses manifested that humoral fibulin-3 yielded a pooled sensitivity of 0.62 (95% CI: 0.45–0.77), specificity of 0.82 (95% CI: 0.73–0.89) and AUC of 0.81 in differentiating MPM patients from cancer-free participants (Figure 3A–3C). Meanwhile, the pooled PLR (positive likelihood ratio), NLR (negative likelihood ratio), and DOR (diagnostic odds ratio) were estimated to be 3.44 (95% CI: 2.24–5.29), 0.46 (95% CI: 0.31–0.69), and 7.44 (95% CI: 3.63–15.25), respectively, corresponding to a diagnostic score of 2.007 (95% CI: 1.290–2.724) (Table 3).

**Table 3:** The pooled analyses of diagnostic efficacy of fibulin-3 in confirming MPM

Analysis	AUC	Pooled sensitivity (95% CI)	Pooled specificity (95% CI)	Pooled DOR (95% CI)	Pooled PLR (95% CI)	Pooled NLR (95% CI)
Overall (adjusted)	0.81	0.62 (0.45–0.77)	0.82 (0.73–0.89)	7.44 (3.63–15.25)	3.44 (2.24–5.28)	0.46 (0.31–0.70)
Overall (unadjusted)	0.74	0.61 (0.46–0.74)	0.76 (0.61–0.87)	5.03 (2.12–11.95)	2.58 (1.47–4.51)	0.51 (0.35–0.75)
Semen type						
Plasma	0.69	0.54 (0.50–0.58)	0.77 (0.74–0.80)	3.55 (2.01–6.27)	2.03 (1.39–2.97)	0.68 (0.51–0.89)
Serum	0.92	0.77 (0.71–0.83)	0.85 (0.79–0.90)	29.11 (9.66–87.74)	5.46 (2.84–10.51)	0.20 (0.07–0.56)
Ethnicity						
Australian	0.59	0.40 (0.35–0.46)	0.78 (0.73–0.82)	2.45 (1.44–4.17)	1.85 (1.13–3.04)	0.83 (0.77–0.90)
American	0.80	0.71 (0.66–0.75)	0.76 (0.73–0.79)	7.44 (3.55–15.60)	2.61 (1.70–4.02)	0.39 (0.26–0.59)
European	0.91	0.75 (0.68–0.81)	0.84 (0.78–0.90)	25.46 (5.51–117.71)	5.23 (2.10–13.06)	0.22 (0.06–0.83)

Abbreviations: CI, confidence interval; PLR, positive likelihood ratio; NLR, negative likelihood ratio; DOR, diagnostic odds ratio; AUC, area under the curve.

Fagan's plot assay displayed apparent improvements of post-test probabilities in the pooled analysis, with a post-test probability of positive result of 46% and negative result of 10% (Figure 3D).

### Subgroup analyses and meta-regression

Subgroup analyses were stratified in terms of test matrix and study ethnicity. The analysis of fibulin-3 efficacy on sample type revealed that serum-based assay harbored higher diagnostic accuracy than plasma-based analysis (sensitivity: 0.77 vs. 0.54; specificity: 0.85 vs. 0.77; DOR: 29.11 vs. 3.55; AUC: 0.92 vs. 0.69). Additionally, analysis based on ethnicity also showed that testing of fibulin-3 in Europeans sustained better efficacy than those in Americans and Australians (Table 3).

Sources of heterogeneity were deeply explored by the univariate meta-regression test with the predefined variations included test matrix, ethnicity, cut-off value, QUADAS score, patient and control sizes, and so forth [22]. As exemplified in Supplementary Table 2, the test matrix (*P* = 0.0052) and control size (*P* = 0.0207) were more likely to be the main causes of heterogeneities among studies.

### Prognosis

**Figure 4:**
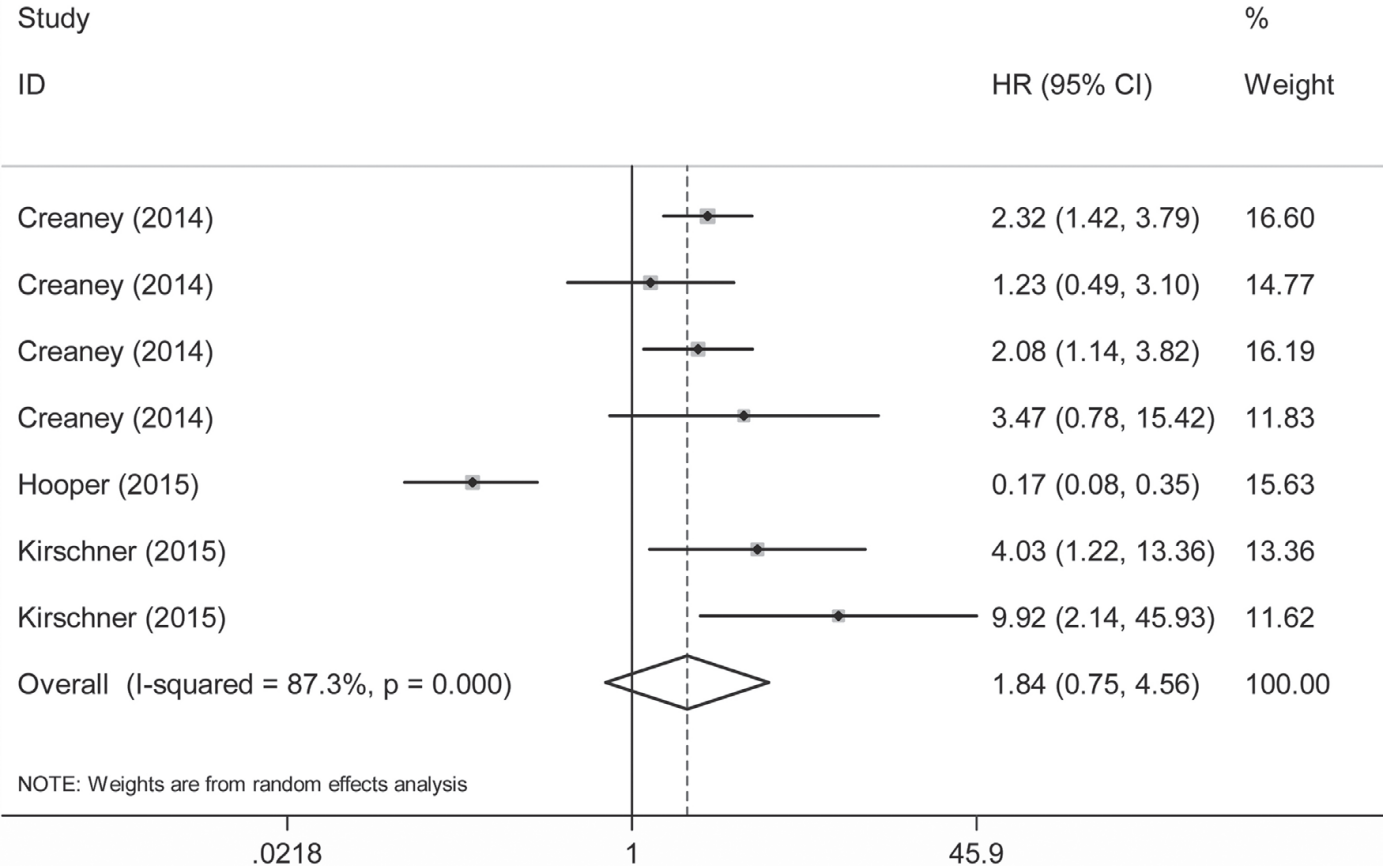
Forest plot of pooled HR for humoral fibulin-3 expression and overall survival (OS) time in MPM patients

Association between fibulin-3 expression and overall survival (OS) time was investigated in 3 studies [10, 15, 16]. As shown in Figure 4, fibulin-3 level was not significantly associated with OS time in MPM patients (HR: 1.84, 95% CI: 0.75–4.56, *P* = 0.185).

### Publication bias

**Figure 5:**
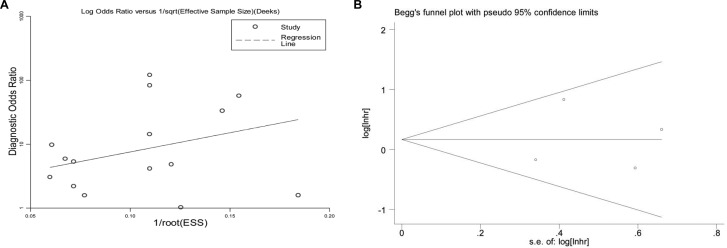
Publication bias assessed by (**A**) Deeks’ funnel plot asymmetry test and (**B**) Bgger's funnel plot.

The *P* values of Deeks’ funnel plot asymmetry test and Egger's tests were estimated to be 0.749 and 0.947, respectively, while Bgger's test also yielded a corrected z value of 0.734 (indicating *P* > 0.05), suggesting that no significant publication bias existed in the meta-analyzed studies (Figure 5 and Supplementary Figure 2).

## DISCUSSION

Malignant pleural mesothelioma (MPM) is one kind of asbestos-related and highly aggressive neoplasm evolves with a median life expectancy of 6–18 months [3, 6]. Patients suffered from MPM often present with non-specific symptoms and therefore result in delayed diagnosis and late-stage disease [1–4]. In 2002, a remarkable research reported that plasma fibulin-3 allowed for the discrimination of MPM patients from cancer-free individuals with high and promising diagnostic efficacy, and therefore strongly recommended its clinical application as a robust indicator in confirming MPM [12]. Since this report, an increasing number of investigations have focused on the diagnostic and prognostic utilities of humoral fibulin-3 in identifying or monitoring MPM [9–18]. Notwithstanding, to our knowledge and available literature, the published data of humoral fibulin-3 testing revealed a high volatility among studies and as yet remains a problem of controversy. We therefore conducted this meta-analysis and made a comprehensive evaluation of the clinic utility of humoral fibulin-3 for MPM diagnosis and prognosis.

Results from our analysis showed that humoral fiulin-3 harbored a pooled sensitivity of 0.62 at a specificity of 0.82 after the outlier-adjustment. The SROC curve presents a comprehensive evaluation of a diagnostic test performance. In our study, the SROC curve analysis displayed an AUC value of 0.81 for humoral fibulin-3 testing, revealing an overall moderate efficacy to aid in MPM diagnosis. Moreover, the diagnostic odds ratio (DOR), was evaluated to be 7.44, showing a relatively high discriminatory performance of fiulin-3 in confirming MPM [23]. In addition, the PLR value of 3.44 suggested that patients with MPM harbored more than 3-fold higher chance of being fibulin-3 positive as compared to the non-MPM individuals. Furthermore, the NLR of fiulin-3 testing was estimated to be 0.46, indicating that fibulin-3 measurement yielded a false-negative rate of 46%, which seems not low enough to exclude MPM. For the clinical utility, testing of fibulin-3 raised the post-test probability of positive result to 46% and lower the post-test probability of negative result to 10% upon a setting of pre-test probability to 20%. Overall, our data demonstrated that fibulin-3 measurement can be popularized as an auxiliary biomarker in identification of MPM.

In the prognostic meta-analysis, only 3 studies were identified [10, 15, 16]. The data revealed that altered expression status of fibulin-3 was not markedly associated with prolonged or shortened survival of MPM (HR: 1.84, 95% CI: 0.75–4.56, *P* = 0.185). However, due to the significant heterogeneity among the pooled analyses as well as the limited sample size and study programs, the results may not completely mirror the actual value of fibulin-3 for prognosis. Further investigations are still warranted to testify our findings.

We further conducted stratified analysis according to different test matrix and study ethnicity. The analysis of fibulin-3 efficacy on sample type revealed that serum-based testing harbored higher diagnostic accuracy than that of plasma-based analysis. Supporting study from Wang et al. demonstrated that the coagulation process is likely to affect the spectrum of extracellular molecules in the blood, hinting that different matrix as serum or plasma may retain altered diagnostic efficacy [24]. Additionally, stratified studies based on ethnicity also showed that testing of fibulin-3 in Europeans achieved superior efficacy than those in Americans and Australians. Nevertheless, the analysis stratified by ethnicity yielded small study size: each analysis only enrolled 2 individual studies and displayed high heterogeneity among studies. Hence, more investigations are still warranted to reinforce this preliminary evidence.

Heterogeneity mainly derives from threshold and non-threshold effects [25–27]. In our study, the Spearman correlation coefficient was applied to trace heterogeneity from threshold effect, yet the Cochran's-Q and I^2^ tests were performed to judge heterogeneity from non-threshold effect. It seemed that heterogeneity caused by threshold only existed in 2 stratified studies (plasma and Australian-based analyses), whereas significant heterogeneity from non-threshold effect appeared in most of our pooled analyses. The different cut-off value or threshold settings contributed the causes of threshold effect [26]. In fact, this is exactly what our data showed: different studies employed different cut-off values varied from 29 to 346 ng/ml for humoral fibulin-3 test. On the other hand, the different examine or reference methods as well as disease conditions and other concomitant diseases contribute to the sources of non-threshold effect [27]. In our study, although all fubulin-3 tests were realized by the employment of ELISA, the control sources were complicated, along with unclear concomitant disease conditions among patients. Results from our meta-regression test showed that the diverse test matrices and limited control sizes were likely to be the major causes of study heterogeneity.

In conclusion, the current meta-analysis suggests that humoral fibulin-3 is acceptable to be a diagnostic biomarker for MPM. Nevertheless, our study still yielded some limitations, such as the population bias, complicated control sources as well as the small case numbers in the stratified analyses. Further studies are still warranted to more comprehensively investigate the prognostic role of humoral fibulin-3 in MPM.

## MATERIALS AND METHODS

### Search strategy and study selection

This meta-analysis was conducted in compliance with the Preferred Reporting Items for Systematic Reviews and Meta-Analyses statement (PRISMA) [28]. Two reviewers independently searched the online PubMed database until June 30th, 2016. The keywords for search were termed as “fibulin-3′’, “mesothelioma/ pleural mesothelioma /malignant pleural mesothelioma”, “sensitivity/specificity/diagnosis/accuracy/ROC” and “prognosis/survival/HR/hazard ratio”. We also manually searched the article references for study identification.

The inclusion criteria were: (1) studies addressed the diagnostic performance of fibulin-3 for MPM identification; (2) studies with sufficient data to establish the 2 × 2 table; (3) studies give a clear definition of the control types; and (4) studies give a calculated HR with 95%CI. The exclusion criteria were: (1) studies failed to clearly definite the control sources; (2) data from studies were insufficient to generate the 2 × 2 table; (3) studies with insufficient data for extracting HR; and (4) non-English articles (full text), letters, duplicate reports, review articles and conference papers, etc.

### Data extraction and article quality assessment

Two reviewers extracted the data with the predesigned extraction forms included the first author, year of publication, country, patient/control size, control types (healthy people or non-cancer cases), sample types (plasma, serum or other), test method, cut-off value, sensitivity and specificity, PFS (progression-free survival), and OS, etc. For the two-stage study contains both training and validation cohorts, data of each group were regarded as independent. Any disagreement was solved by group discussion.

Article quality was judged based on the Quality Assessment for Studies of Diagnostic Accuracy (QUADAS) II and Newcastle-Ottawa Scale (NOS) checklists [20, 21].

### Statistical analyses

Statistical analyses were undertaken based on the platforms of two statistical software programs: Stata 12.0 (Stata Corporation, College Station, TX, USA) and Meta-disc 1.4 (XI Cochrane Colloquium, Barcelona, Spain). For the diagnostic meta-analysis, a bivariate model allowed for the plotting of a summary receiver operating characteristic (SROC) curve as well as the pooled sensitivity, specificity, PLR, NLR and DOR. In the prognostic analysis, the HR and its 95% CI were extracted for aggregation of the survival results. Heterogeneity from the threshold effect was evaluated by the Spearman correlation coefficient, and *P* < 0.05 indicates the existence of significant heterogeneity [22]. Cochran's-Q test and I^2^ test were applied to detect heterogeneity from non-threshold effects (*P* < 0.01 or I^2^ > 50%) [22]. Moreover, influence analysis and meta-regression test were employed to trace the potential sources of study heterogeneity. Publication bias was examined by Deek's funnel plot asymmetry test (for diagnostic studies), and Bgger's and Egger's funnel plots (for prognostic studies).

## SUPPLEMENTARY MATERIALS FIGURES AND TABLES


