# Correction: Diagnostic and prognostic utilities of humoral fibulin-3 in malignant pleural mesothelioma: Evidence from a meta-analysis

**DOI:** 10.18632/oncotarget.25301

**Published:** 2018-04-20

**Authors:** Dongxu Pei, Yongwei Li, Xinwei Liu, Sha Yan, Xiaolan Guo, Xiaona Xu, Xiaoxia Guo

**Affiliations:** ^1^ Department of Clinical Laboratory, Henan Province Hospital of TCM, Zhengzhou, Henan, China; ^2^ Department of Clinical Laboratory, Anyang Hospital of Traditional Chinese Medicine

**This article has been corrected:** The correct author affiliation is given below:

^1^ Department of Clinical Laboratory, Henan Province Hospital of TCM, Zhengzhou, Henan, China

Original article: Oncotarget. 2017; 8:13030-13038. https://doi.org/10.18632/oncotarget.14712

