# Supplementary material for: Diagnostic and prognostic utilities of humoral fibulin-3 in malignant pleural mesothelioma: Evidence from a meta-analysis
Source: Oncotarget. 2017 Jan 18;8(8):13030–8. doi: 10.18632/oncotarget.14712 (PMC5355074; doi:10.18632/oncotarget.14712)
Supplement: Supplementary file 1 [file oncotarget-08-13030-s001.pdf]

Diagnostic and prognostic utilities of humoral fibulin-3 in malignant pleural mesothelioma: Evidence from a meta-analysis

Supplementary Materials

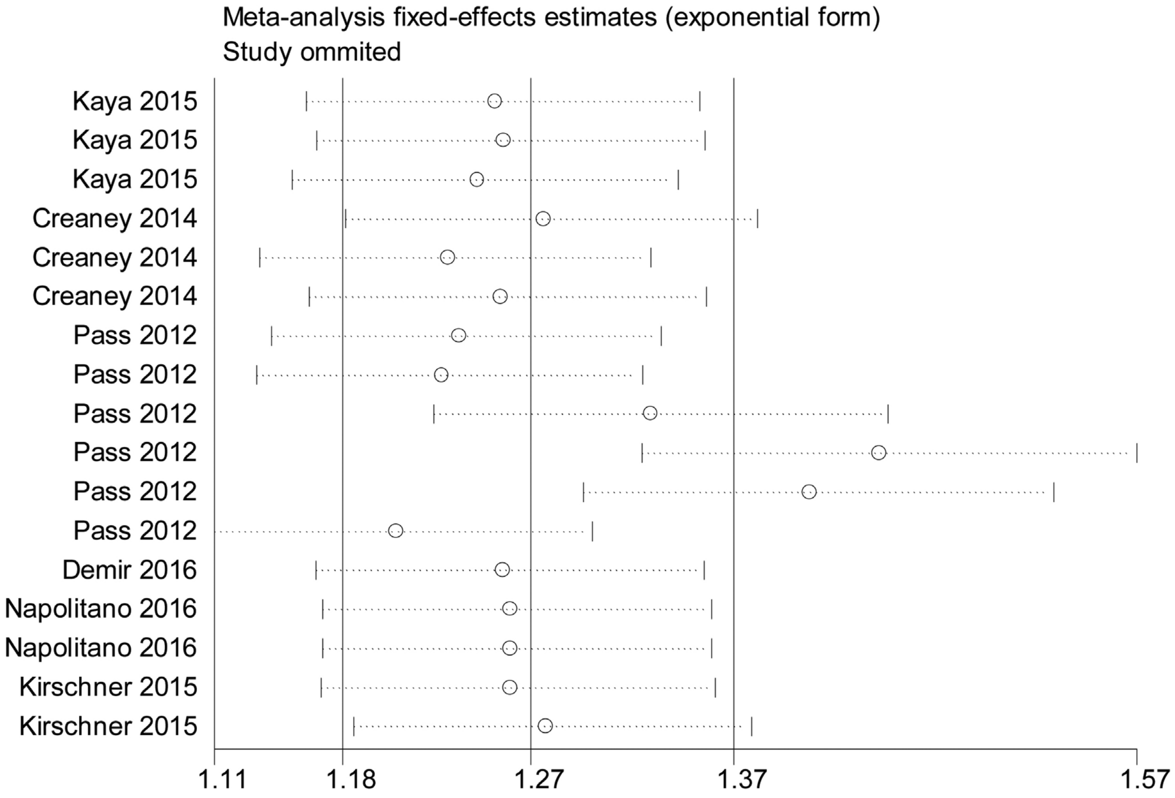

Supplementary Figure 1: Influence analysis of the outliers among enrolled studies.

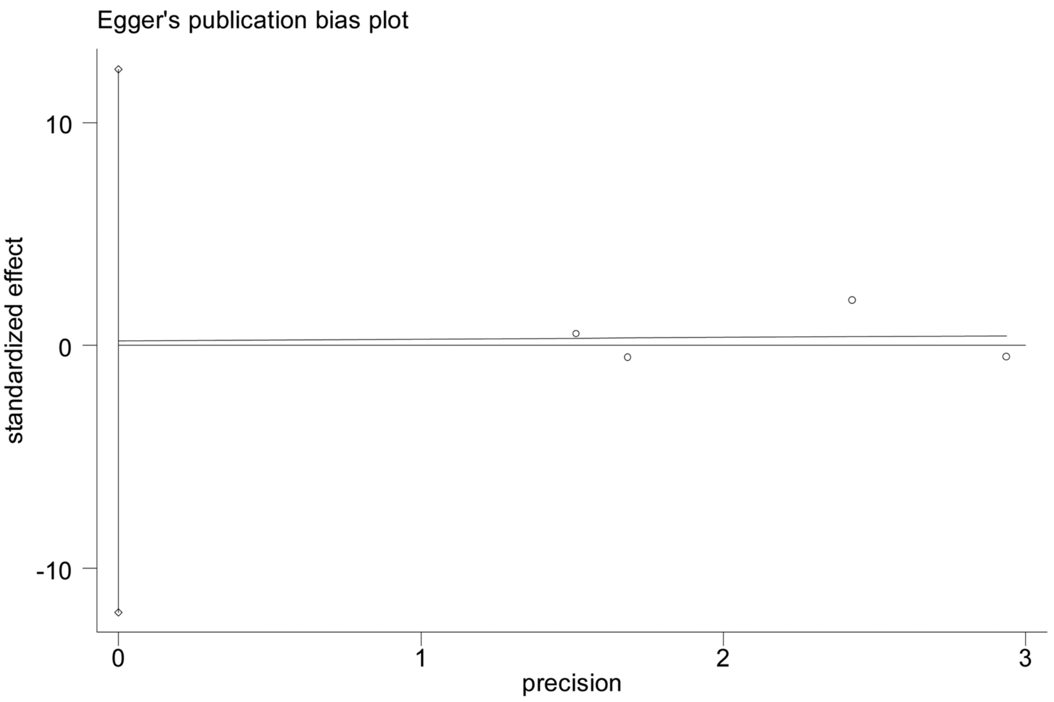

Supplementary Figure 2: Analysis of the publication bias by Egger linear regression test.

Supplementary Table 1: Summary of humoral fibulin-3 used as prognostic biomarker of MPM

Author	Year	Area	MPM number	Study design	Sample type	Test method	Survival analysis	HR availability	Follow-up months	NOS score
Creaney et al. [10]	2014	Australia	80	Retrospective	Plasma and effusion	ELISA	OS	Directly	20	8
Kirschner et al. [15]	2015	Australia	30	Retrospective	Effusion	ELISA	OS	Directly	> 12	7
Hooper et al. [16]	2015	UK	58	Retrospective	Serum	ELISA	OS	Directly	> 12	7

Supplementary Table 2: Exploration of the potential sources of heterogeneity by meta-regression test

Study characteristic	*P* value	RDOR	95% CI
Test matrices (Serum vs. Plasma vs. Pleural effusion)	0.0052	0.13	(0.03–0.49)
Study ethnicity (Australian vs. American vs. European)	0.9332	1.07	(0.18–6.20)
MPM cases (< 100 vs. ≥ 100)	0.0961	18.08	(0.55–589.89)
Control size (Control < 100 vs. Control ≥ 100)	0.0207	6.34	(1.40–28.76)
Cut-off value (< 50 vs. OD ≥ 50 vs. unclear)	0.9042	0.94	(0.29–2.99)
Article quality (QUADAS scores)	0.6373	0.74	(0.19–2.85)

MPM: malignant pleural mesothelioma; RDOR: relative diagnostic odds ratio; CI: confidence interval; QUADAS: quality assessment for studies of diagnostic accuracy.
